# Inverse association of anti-inflammatory prescription fills and suicide-related mortality in young adults: Evidence from a nationwide study of Swedish regions, 2006–2021

**DOI:** 10.1016/j.bbih.2023.100665

**Published:** 2023-06-28

**Authors:** Peter Andersson, Johan Lundberg, Håkan Jarbin, Jussi Jokinen, Adrian E. Desai Boström

**Affiliations:** a Department of Clinical Neuroscience/Psychology, Karolinska Institutet, Stockholm, Sweden; b Centre for Clinical Research, Falun, Uppsala University, Sweden; c Stockholm Health Care Services, Region Stockholm, Sweden; d Centre for Psychiatry Research, Department of Clinical Neuroscience, Karolinska Institutet, & Stockholm Health Care Services, Region Stockholm, Karolinska University Hospital, SE-171 76, Stockholm, Sweden; e Child and Adolescent Psychiatry, Region Halland, Sweden; f Department of Clinical Sciences Lund, Section of Child and Adolescent Psychiatry, Lund University, Lund, Sweden; g Department of Clinical Sciences/Psychiatry, Umeå University, Umeå, Sweden

## Abstract

**Background:**

This cross-sectional study examined nationwide real-world associations between anti-inflammatory agent fills and suicide-related death rates in 20–24-year-olds across the 21 Swedish regions during 2006–2021.

**Methods:**

Nationwide Swedish registers were used to compare regional year-wise suicide-related mortality (SRM) and dispensations for anti-inflammatory agents (ATC-code: M01) in 20–24-year-olds. Dispensations for paracetamol (ATC-code: N02BE01) was applied as a control variable. Associations between regional year-wise SRM and dispensation rates were analyzed by sex-stratified zero-inflated generalized linear mixed effect models (GLMM). Dispensation rates of paracetamol and inflammatory agents were designated as independent fixed effects variables, and year and region constituted random-intercept effects.

**Results:**

Acetic acid derivatives and related substances (M01AB) and propionic acid derivates (M01A3) accounted for ∼71% of measured dispensation fills for anti-inflammatory agents. Diclofenac fills constituted ∼98% of the former category, whereas dispensations for Ibuprofen (∼21%), Naproxen (∼62%) and Ketoprofen (∼13%) constituted the most prescribed agents in the latter category. Regional yearly dispensation rates of anti-inflammatory agents in 20–24-year-old females were inversely associated with female SRM (β = −0.095, *p* = 0.0393, 95% CI -0.186, −0.005) – independent of paracetamol rates, which were unassociated to SRM (p = 0.2094). Results were confirmed in validation analyses for anti-inflammatory agents (OR = 0.7232, *p* = 0.0354, 95% CI [OR] 0.5347, 0.9781). No association was demonstrated in males (*p* = 0.833).

**Conclusion:**

Anti-inflammatory agent dispensation rates were independently associated to lower suicide-related death rates in female 20-24-year-olds. This adds to growing evidence implicating inflammatory processes in mental disorders, warranting trials focusing on the suicide preventative potential of anti-inflammatories in young adults.

## Highlights

• 2006–2021 study across the 21 Swedish regions encompassing 3453 suicide-related deaths.
• Female SRM inversely correlated with anti-inflammatory agent use (p = 0.0393).
• Validation confirms anti-inflammatory agents' suicide risk reduction (p = 0.0354).
• Study supports link between inflammation & mental disorders.
• Trials of anti-inflammatories for suicide prevention in young adults warranted.

## 1 Introduction

Suicide constitutes a major global health challenge, estimated to cause approximately 800,000 deaths per annum (World Health Organization (WHO), 2019). Mortality due to suicide have declined during recent decades (Naghavi, 2019a), but is still the leading cause of death worldwide among 15-24-year-olds (Fazel et al., 2020). Over the whole life-span, suicide deaths occurs more frequently in males. Data from the Global Disease Burden Study suggest that this pattern is established in young adulthood, with estimated global annual mortality rates of 8.5 and 8.2 per 100,000 in females and males, respectively, in the 15–19 age-span, compared to 10.2 and 16.2 per 100,000 in young adulthood (20–24 age span) (Naghavi, 2019b). Major depressive disorder (MDD) may be the most important contributor to confirmed suicide deaths in adolescence and young adulthood, according to psychological autopsy studies indicating the presence of a mood disorder in 44–76% of unselected suicide victims in this age group (Bridge et al., 2006a). One main hypothesis on the patohysiology of MDD involves neuroinflammation and elevated concentrations of several inflammatory markers have been observed in plasma of depressed individuals (Ye et al., 2021; Hannestad et al., 2011; Kim et al., 2021). Batty et al. presented evidence from a large UK cohort study, demonstrating an association between elevated levels of the inflammatory marker serum C-reactive protein (CRP) and subsequent suicide deaths (Batty et al., 2016). However, the current evidence in support of an association between inflammation and depression in children and adolescents appears inconclusive (Colasanto et al., 2020; D'Acunto et al., 2019). For example, recent works by Iob et al. could not demonstrate any associations between individual nor longitudinal patterns of CRP from childhood to adolescence (9-18-year-olds) and depressive symptoms in young adulthood (20-23-year-olds), positing that early-life inflammatory markers may be unrelated to the etiology of adult-onset depression (Iob et al., 2022). The authors suggest that the biological relevance of inflammation might be different between childhood/adolescence and adulthood. The involvement of inflammation in early adulthood MDD has, however, not been reliably disputed. On the contrary, Kim et al. recently demonstrated that peripheral inflammatory biomarkers in drug-naïve patients with MDD in young adulthood may be associated with the clinical symptoms of MDD, including depressive moods, hopelessness, suicidal ideation, low self-esteem and reduced psychological resilience (Kim et al., 2021). The current evidence base would thus suggest that the association between inflammation and depression could be more robustly present in early adulthood than in other age groups. Indeed, markers of inflammation were not associated with later-life depression in an independent study of older subjects (mean age 57.3–65.2) screened to exclude physical illness (Luning Prak et al., 2022). Interestingly, early adulthood (20–24 age-range) also constitutes the peak age of onset of major depressive disorder (MDD) (Solmi et al., 2021).

The antidepressant effects of anti-inflammatory medication, either as stand-alone or adjuvant treatment to traditional antidepressant pharmacotherapy, has been investigated in several trials. In a meta-analysis, Köhler et al. (2014) included ten trials investigating the antidepressant effects of non-steroidal anti-inflammatory drugs (NSAIDs) in adults (age range:18–70) - four of which as adjuvant treatment, and six as monotherapy. Four additional trials investigating the antidepressant effects of cytokine inhibitors as monotherapy were also included. The study population included both individuals with depressive symptoms and those fulfilling MDD-criteria. Support for antidepressant effects of anti-inflammatory treatments was demonstrated, with sub-analyses indicating large increases in the likelihood of both remission (4 studies, 132 patients: OR = 7.89, 95% CI: 2.94–21.17) and response (3 trials, 92 patients: OR = 6.59, 95% CI: 2.24 to 19.42) in patients who received the selective NSAID celecoxib as adjuvant antidepressant treatment. However, studies reporting longer follow-up times than 6–12 weeks were rare and only one trial spanned a full year. Additionally, substantial heterogeneity and high risk of bias were factors limiting the results (Köhler et al., 2014). A later meta-analysis of 14 studies (one study reported inclusion of <18 year-olds) by Husain et al. provided preliminary evidence for anti-manic and anti-depressive effects of anti-inflammatory medication, both as stand-alone and adjuvant treatment (Husain et al., 2017). However, the population analyzed included patients with both MDD and bipolar disorder, arguably two heterogeneous conditions. Similarly to Köhler et al. (2014), the limitations noted by the authors included an absence of longer follow-up times (a majority of studies reported outcomes at 6–12 weeks), rendering it impossible to fully evaluate potential adverse consequences, tolerability and long-term efficacy (Husain et al., 2017). More recently, Bai et al. conducted a meta-analysis of 30 RCTs analyzing anti-inflammatory treatment as mono- and adjunctive therapy in adults (range of mean age in included studies: 30.38–83.91 years) diagnosed with MDD (Bai et al., 2020). Agents studied included celecoxib, statins, minocycline, pioglitazone, modafinil^,^ and N-acetylcysteine. The analysis demonstrated effectiveness in reducing depression severity from both mono- and adjuvant anti-inflammatory therapy. Treatments were shown as safe and generally well-tolerated, although short treatment periods precluded any evaluation of adverse consequences and efficacy in the longer-term.

Inflammation is a possible contributor to MDD and the current evidence-base suggests this association holds strongest in early adulthood. Growing evidence support the safety and efficacy of anti-inflammatory agents for treating MDD. Drawing on more credible evidence that MDD is a major contributor to suicide deaths, the authors hypothesized that dispensation rates of anti-inflammatory drugs could exert an effect on suicide-related death rates at the regional level. Drawing on regional year-wise differences for statistical inferences, the present study investigated associations between regional yearly suicide-related mortality (SRM) in 20–24-year-olds and anti-inflammatory agent prescription fills by generalized linear mixed models. Data was comprehensively retrieved from nationwide Swedish registers, encompassing the 21 Swedish regions during the years 2006–2021. Based on previous research implicating substantial sex differences in suicide-related outcomes (Naghavi, 2019b) and analgesic prescription rates (Bäckryd, 2018), males and females were studied separately.

## 2 Methods

### 2.1 Study population

This register-based cross-sectional study follows the Strengthening the Reporting of Observational Studies in Epidemiology (STROBE) reporting guideline (Elm et al., 2007). Register studies do not require informed consent in Sweden. The study was preregistered via the Open Science Framework on November 28th, 2022 (OSF Registries | Association between anti-inflammatory prescription fills and suicide deaths among young adults in Sweden).

Openly available data from the Swedish National Board of Health and Welfare (available in Swedish: https://sdb.socialstyrelsen.se/if_dor/val.aspx; https://sdb.socialstyrelsen.se/if_lak/val.aspx) was extracted for 21 Swedish counties across 2006–2021 in 20–24-year-olds and for the following variables: The number of dispensations recorded for anti-inflammatory agents (ATC-code M01) per 1000 inhabitants (based on population estimates from January 1st of the recorded year) and dispensations for paracetamol (ATC-code: N02BE01) were retrieved as a control variable. Values for paracetamol and anti-inflammatory agent dispensation rates were multiplied by 100 to arrive at a representative estimate per 100,000 inhabitants. The cause-of-death register was used to extract suicide-related death rates per 100,000 inhabitants (ICD-10 registry: X60-X84 – intentional self-harm, and Y10–Y34 – event of undetermined intent). All data was extracted for males and females, separately – across all Swedish counties for the years 2006–2021. The time-period included all the years for which such data was openly available. No data points were excluded and there were no missing data points.

### 2.2 Statistical considerations

Several measures were taken with the aim of strengthening robustness of included variables. These initial steps were performed in using Microsoft Excel for Microsoft 365 MSO (Version 2204 Build 16.0.15128.20278).

### 2.3 Variable distribution and model prespecifications

The distribution of the variables was investigated by Shapiro-wilks tests and visually inspected from histogram and Cullen and Frey plots (Delignette-Muller and fitdistrplus, 2015; Cullen et al., 1999). No variable fully satisfied criteria for normal distribution. It was determined upon visual inspection of Cullen and Frey plots that the response variable could be modeled on the general poisson distribution in the case of females, and, for males, the gamma distribution (Ctr et al., 2011). Moreover, the data set included a reasonable amount of zeros (i.e., 39/336∼11.6% for males and 144/336∼42.8% for females) with large regional differences. For data exhibiting such characteristics, Favero et al. recently demonstrated the importance of considering random effects at the regional level and the zero-inflated nature of the outcome variable (Fávero et al., 2021). Hence, it was determined that the analyses should be modeled on the zero-inflated gamma distribution for males, and the zero-inflated poisson distribution in the case of females. We used glmmTMB models due to their increased flexibility in managing zero-inflated data and its higher speed when using multiple fixed effects as well as random effects (Brooks et al., 2017a).

### 2.4 Measures to reduce potential unmeasured confound

Confound could arise from region-specific effects regarding anti-inflammatory agent dispensation rates and suicide-related death rates. For example, regional variations in population size, socioeconomic status, substance abuse, or availability and quality of psychiatric care, could exert distorting effects on the extracted variables. To reduce any distorting effects on our results from such potential sources of confound, paracetamol and anti-inflammatory agent dispensation rates in 20–24-year-olds were adjusted for as fixed effects variables, and region and year as random-intercept effects. Moreover, basing the analysis on the number of dispensations should reduce influence from observations of short-term treatment (compared to studying the number of individual patients receiving treatment) and also better account for patient adherence to prescribed agents (compared to exclusively depending on the number of individual patients receiving treatment). In summarizing, the data entailed sex-stratified regional rates (i.e., 21 regions, 16 years and 2 sexes = 672 total observations or 336 observations for each sex, respectively). Sex-stratified regional year-wise suicide-related death rates in 20–24-year-olds were defined as outcome variables, paracetamol and anti-inflammatory agent dispensation rates in 20–24-year-olds as exposures, and year and region as effect (intercept) modifiers.

### 2.5 Statistical analysis

**Supplemental Fig. 1:**
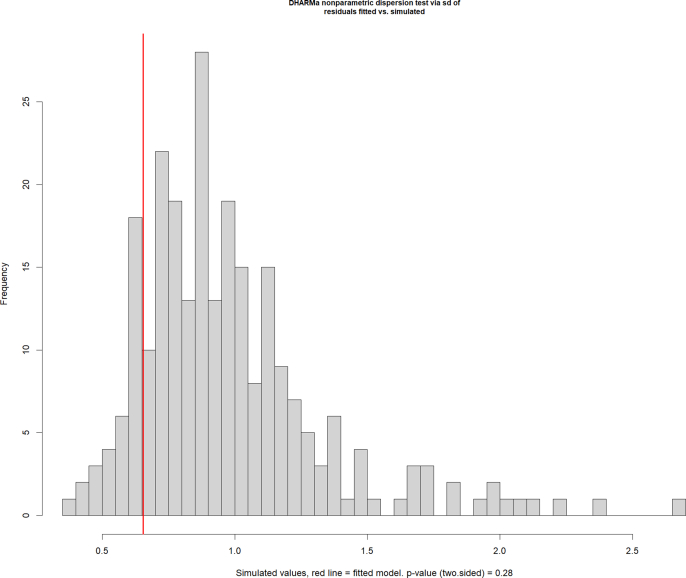
Title: DHARMa test for dispersion, before correction.

**Supplemental Fig. 2:**
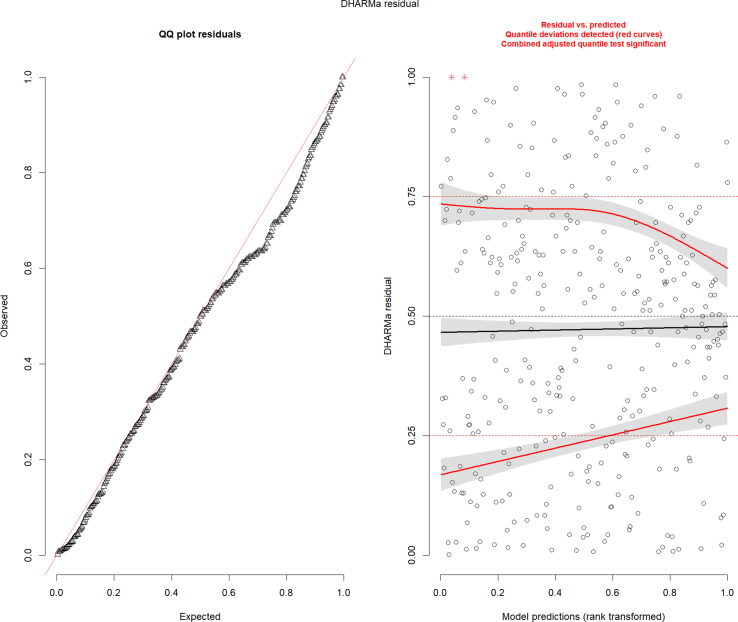
Title: QQ-plots residuals and residuals vs. predicted lines, before correction.

**Supplemental Fig. 3:**
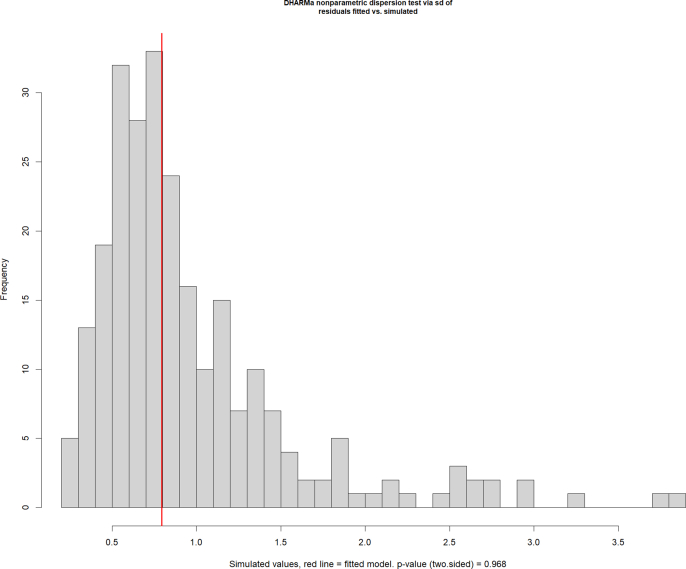
Title: DHARMa test for dispersion, after correction.

**Supplemental Fig. 4:**
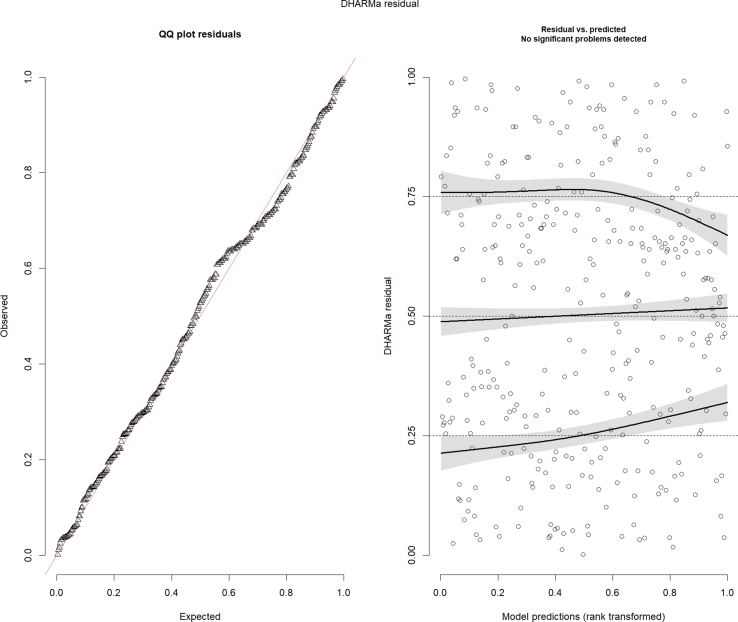
Title: QQ-plots residuals and residuals vs. predicted lines, after correction.

**Supplemental Fig. 5:**
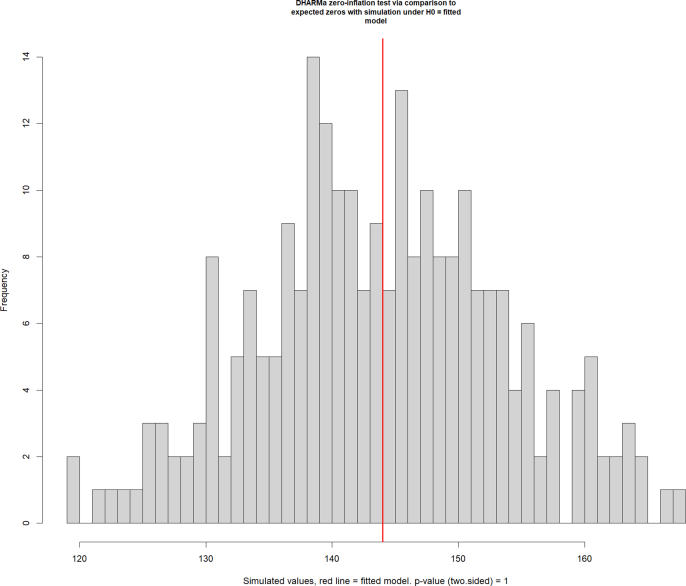
Title: Zero-inflation test via comparison to expected zeros, after correction.

All statistical analyses were performed in using R version 4.0.3. The variables included in the analysis pertained to Swedish 20-24-year-olds in the years 2006–2021 and included year-wise regional paracetamol and anti-inflammatory agent dispensation rates, and suicide-related death rates. Exposure variables (regional year-wise paracetamol and anti-inflammatory agent dispensation rates in 20–24-year-olds) were investigated for normal distribution (Shapiro-Wilks test) and collinearity (Pearson correlations) in each sex-group, separately. None of the exposure variables satisfied requirements for normal distribution and were thus subjected to transformation by Blom's method for all subsequent analyses. Collinearity was not evident in either females (*r* = 0.04) or males (*r* = 0.15). Thus, the exposure variables were treated as independent in downstream analyses. Associations between the outcome variable (regional year-wise SRM), exposures (Blom-transformed values for paracetamol and anti-inflammatory agent dispensation rates in 20–24-olds, respectively) were investigated separately for each sex by generalized linear mixed effects models, whereby paracetamol and anti-inflammatory agent dispensation rates in 20–24-year-olds were designated as fixed-effects variables, and effect modifiers (region and year) were treated as random-intercepts. The ‘glmmTMB’ package for R-statistics was implemented for these analyses (Brooks et al., 2017b), modeled on the poisson (females) and gamma (males) distribution. P-values <0.05 were considered significant. For all models assessing NSAID and paracetamol dispensations in relation to regional year-wise suicide-related death rates, we used likelihood ratio tests using maximum-likelihood estimation to assess the statistical significance of individual variables (i.e., NSAID/Paracetamol dispensation rates, year, region, regional population expressed as a percentage of national population). Specifically, we compared the full model versus a reduced model without the individual variable (single term deletion). The final model included all explanatory variables with residual deviance χ2 < 0.05 (“chi-squared value”) in the model comparison with the full model (i.e., all included variables except for the proportional regional population variable). Significant models were tested for dispersion, heteroscedasticity and zero-inflation. First, we used the DHARMa test for dispersion (‘testDispersion’-function of the DHARMa package for R Statistics (DHARMa, 2022)) to test if main models were under- or overdispersed. No main model exhibited signs of over- or underdispersion by this analysis (p > 0.05) (Supplemental Fig. 1). Second, residuals were illustrated in ‘QQ-plots’ and ‘Residuals vs. predicted values’ plots using the ‘simulateResiduals’-function of the DHARMa (DHARMa, 2022). The exact p-values for the quantile lines in the plot were calculated by the ‘testQuantiles’-function of the same R package – and demonstrated signs of heteroscedasticity in females (Supplemental Fig. 2.). The recommended procedures for addressing heteroscedasticity in our models were strictly followed, as outlined in the ‘DHARMA’ package for R statistics (DHARMa, 2022). To achieve this, a simple overdispersion correction was added as a random effect intercept to the original model suffering from heteroscedasticity. The correction variables were introduced using a principled approach, where the relative regional weight was included as an interaction term to the factor variable ‘Region’ to minimize model adjustments and ensure that results were not affected by heteroscedasticity. To avoid the issue of multicollinearity, the relative weights were not included as an independent variable due to the high correlation between them. Instead, an interaction term between region and proportional regional population was added for female-specific analyses, resulting in a model without signs of overdispersion or heteroscedasticity, as confirmed by the same assessment methods (Supplemental Figs. 3–4.). Lastly, the ‘testZeroInflation’-function of the DHARMa package for R statistics (DHARMa, 2022) confirmed our previous assumption of zero-inflation (Supplemental Fig. 5.).

**Supplemental Fig. 6:**
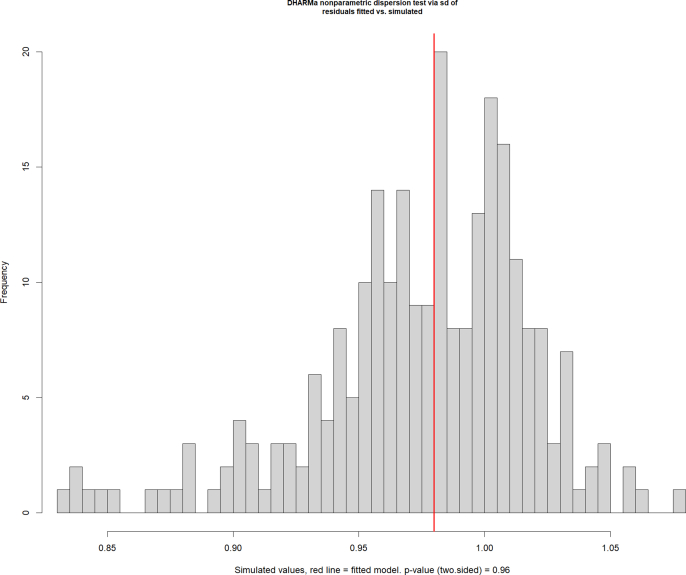
Title:DHARMa test for dispersion in validation analyses.

**Supplemental Fig. 7:**
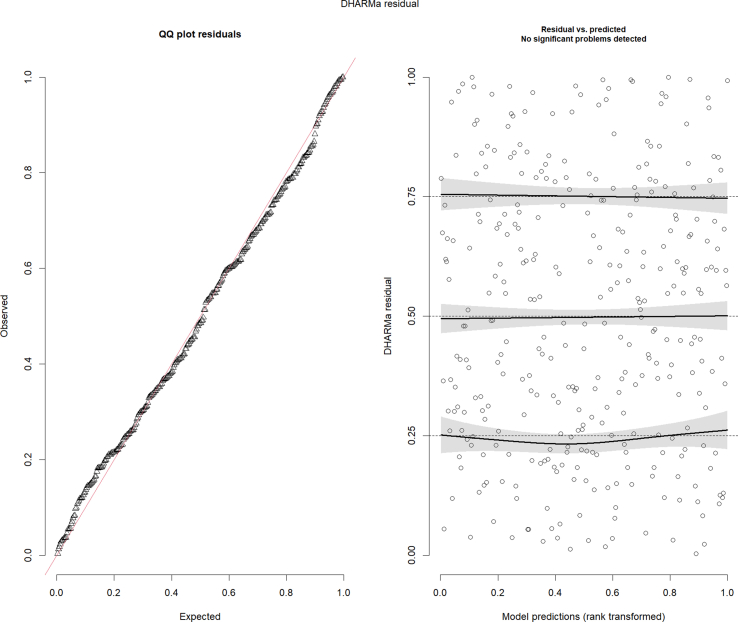
Title:QQ-plots residuals and residuals vs. predicted lines in validation analyses.

For validation purposes, significant models were further explored by separate analyses. In these post-hoc analyses, we first dichotomized suicide-related death rates based on the 75th percentile – whereby top-quartile observations regarding suicide-related death rates were compared to Q1-Q3 observations. To account for the binomial distribution of the dichotomized response variable, generalized linear mixed effects models modeled on the beta binomial distribution were implemented. Similarly, year and region were treated as random-intercept effects. P-values <0.05 were again considered significant. Significant validation models were tested for dispersion and heteroscedasticity by the same methods as described above. As no such signs were evinced (Supplemental Figs. 6–7.), the proportional regional population variable was not included in the validation analyses. To investigate the potential effect of unaccounted for confound arising from differences in regional year-wise prevalence rates of somatic health diagnoses, any associations between NSAID prescription rates and SRM were further validated in additional post-hoc analyses. In these analyses, additional adjustments were made for the regional year-wise diagnosis rate of ‘unspecified pain’ (ICD-10 code: R52), to serve as a control proxy variable for the burden of pain-related conditions that also may have an influence on analgesic prescription rates and SRM.

## 3 Results

### 3.1 Baseline characteristics of data

**Fig. 1:**
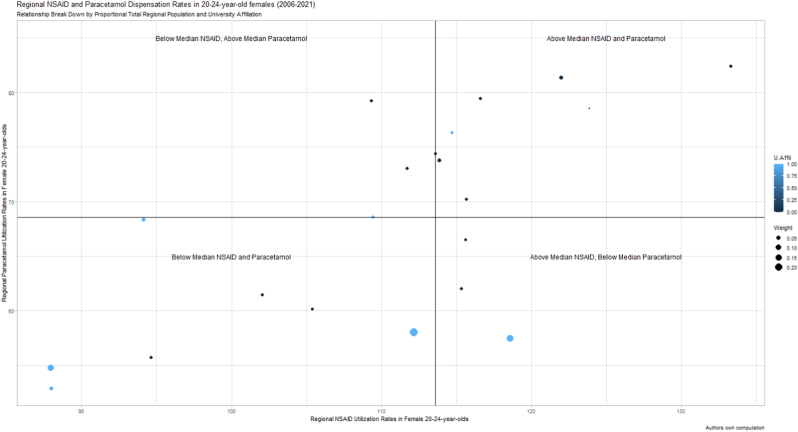
Regional NSAID and Paracetamol Dispensation rates in 20–24-year-old females (2006–2021) **Figure Legend (**Fig. 1**):** The y-axis illustrates the median yearly regional paracetamol dispensation rate in Swedish 20-24-year-old females across 2006–2021. Similarly, the x-axis illustrates the median yearly regional NSAID dispensation rate in Swedish 20-24-year-old females across 2006–2021. The straight lines illustrate the national median for the same age-group and time-period. University affiliated counties are highlighted in blue, others in black. County population, retrieved from the Swedish Central Bureau of Statistics, is illustrated by circle diameter. (For interpretation of the references to colour in this figure legend, the reader is referred to the Web version of this article.)

**Fig. 2:**
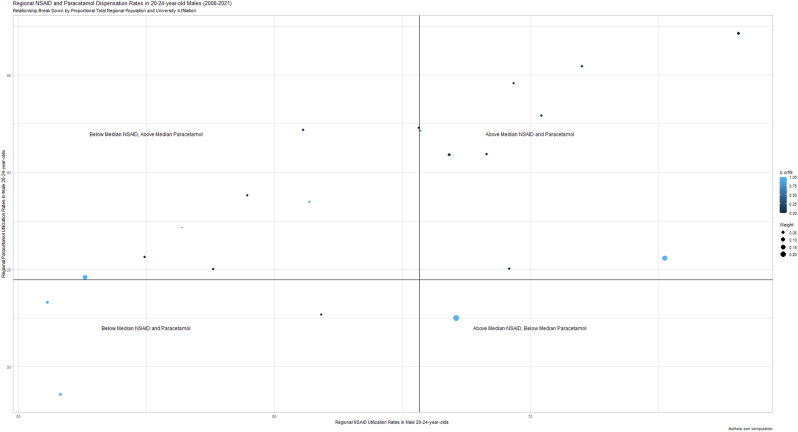
Regional NSAID and Paracetamol Dispensation rates in 20–24-year-old males (2006–2021) **Figure Legend (**Fig. 2**):** The y-axis illustrates the median yearly regional paracetamol dispensation rate in Swedish 20-24-year-old males across 2006–2021. Similarly, the x-axis illustrates the median yearly regional NSAID dispensation rate in Swedish 20-24-year-old males across 2006–2021. The straight lines illustrate the national median for the same age-group and time-period. University affiliated counties are highlighted in blue, others in black. County population, retrieved from the Swedish Central Bureau of Statistics, is illustrated by circle diameter. (For interpretation of the references to colour in this figure legend, the reader is referred to the Web version of this article.)

The sample comprehensively encompassed all registered Swedish citizens that in the years 2006–2021 were aged 20-24-years-old and who died by a suicide-related event or were prescribed paracetamol or an anti-inflammatory agent (stratified by region and sex) – in a sample encompassing 1657 deaths attributed to suicide (n = 1310) and damage events with unclear intent (n = 347). Acetic acid derivatives and related substances (M01AB) and propionic acid derivates (M01A3) accounted for ∼71% of measured dispensation fills for the anti-inflammatory agent variable. Diclofenac fills constituted ∼98% of the former category, whereas dispensations for Ibuprofen (∼21%), Naproxen (∼62%) and Ketoprofen (∼13%) constituted the most prescribed agents in the latter category. Mean regional NSAID and paracetamol dispensation rates over 2006–2021 are illustrated in Fig. 1, Fig. 2. There were substantial differences between the sexes regarding mean national suicide-related death rates across 2006–2021, with males overrepresented by factor ∼2 (i.e., for females, mean 9.7 [SD: 1.6] and, for males, 23.6 [SD: 2.7]). Extracted data is summarized in Table 1.

**Table 1:** Yearly national rates of NSAID and paracetamol dispensation, patients diagnosed with pain not otherwise specified (ICD-10 code: R52) and suicide related mortality in males and females aged 20–24, per 100000 inhabitants during 2006–2021.

	2006	2007	2008	2009	2010	2011	2012	2013	2014	2015	2016	2017	2018	2019	2020	2021
**NSAID dispensation rates, males**	8233 (1006.4)	7817 (894.8)	7973 (973.5)	7372 (780.7)	7365 (1014.0)	7332 (1008.4)	7256 (1007.0)	6717 (1011.5)	6609 (913.5)	6312 (996.5)	6074 (938.0)	5797 (843.2)	5733 (1000.1)	5575 (791.7)	5134 (732.0)	5424 (782.7)
**NSAID dispensation rates, females**	12782 (1202.1)	12098 (1208.7)	12290 (1221.9)	11772 (1179.1)	11703 (1167.4)	11781 (1470.1)	11668 (1476.7)	11191 (1252.6)	10897 (1147.1)	10872 (1280.5)	10465 (1237.4)	10021 (1534.0)	9821 (1516.9)	9683 (1389.6)	9849 (1257.2)	10250 (1430.0)
**Paracetamol dispensation rates, males**	2929 (593.6)	3151 (768.5)	3379 (879.2)	3395 (735.8)	3336 (701.5)	3459 (662.0)	3564 (578.1)	3584 (644.5)	3730 (640.5)	3805 (565.4)	4119 (605.2)	4161 (741.1)	3724 (656.3)	3748 (647.9)	3566 (575.9)	3562 (489.0)
**Paracetamol dispensation rates, females**	4205 (733.3)	4318 (827.4)	4854 (842.8)	5148 (1025.1)	5210 (1055.9)	5397 (1079.8)	6049 (1135.1)	6572 (1013.2)	6869 (1184.7)	7384 (1308.0)	7712 (1499.8)	7759 (1577.4)	6559 (1195.6)	6465 (917.1)	6307 (1115.8)	6233 (1148.3)
**Patients with diagnosis of Pain NOS(ICD-10 Code: R52), males**	141.3 (118.6)	163.6 (72.2)	172.8 (72.2)	168.7 (76.9)	180.2 (79.6)	195.3 (87.7)	227.2 (105.8)	257.9 (95.9)	276.6 (116.7)	308.1 (139.0)	308.1 (143.0)	297.0 (134.6)	326.8 (110.2)	305.2 (128.7)	288.5 (141.5)	340.9 (195.7)
**Patients with diagnosis of Pain NOS(ICD-10 Code: R52), females**	170.8 (131.7)	178.9 (94.9)	176.0 (97.8)	181.1 (86.9)	173.5 (83.1)	203.8106.3)	226.7 (99.8)	252.8 (165.2)	277.4 (120.2)	284.1 (119.3)	290.1 (143.3)	286.2 (143.4)	294.0 (181.1)	283.7 (161.7)	250.8 (94.5)	285.2 (109.4)
**Suicide related mortality, males**	21.57 (17.62)	19.54 (17.11)	27.49 (19.94)	25.07 (18.91)	24.35 (16.19)	19.52 (27.69)	24.32 (14.10)	27.42 (18.34)	27.01 (30.15)	21.38 (20.95)	18.83 (11.28)	23.06 (19.85)	24.74 (19.90)	23.8 (18.48)	23.62 (18.35)	26.03 (19.30)
**Suicide related mortality, females**	11.49 (9.82)	8.95 (9.76)	9.00 (10.34)	9.67 (11.48)	7.93 (11.64)	7.01 (9.36)	8.39 (6.24)	11.32 (8.05)	9.46 (10.62)	9.61 (11.52)	10.2 (16.00)	8.57 (7.60)	12.33 (16.42)	13.49 (16.77)	8.74 (13.28)	8.84 (10.72)

**Table Legend (**Table 1**):** Yearly Swedish national rates of NSAID and paracetamol dispensation, incidence of pain not otherwise specified and suicide related mortality per 100,000 inhabitants in 20–24 year old males and females during 2006–2021. Numbers in parentheses are the standard deviations of yearly regional rates in the 21 administrative regions.

### 3.2 Regional year-wise dispensation rates of anti-inflammatory agents are negatively associated with suicide-related death rates in 20–24-year-old females

Regional yearly dispensation rates of anti-inflammatory agents in 20–24-year-old females were inversely associated with female SRM (β = −0.095, p = 0.0393, 95% CI -0.186, −0.005) (Supplemental Figs. 3–5) – independent of paracetamol fill rates, which were unassociated to female SRM (β = −0.056, p = 0.1278) (Table 2.). In validation analyses modeled on the beta-binomial distribution, results were confirmed for anti-inflammatory agent dispensation rates (OR = 0.7232, p = 0.0354, 95% CI [OR] 0.5347, 0.9781) but not for paracetamol (OR = 0.906, p > 0.1) (Table 3.) (Supplemental Figs. 6–7). No association was demonstrated in males (*p* = 0.833).

**Table 2:** Associations between regional year-wise suicide-related mortality in 20–24-year-olds and anti-inflammatory agent dispensation rates.

		Coef.	Std. Error	z value	P-value
Males	(Intercept)	3.28743	0.070122	46.88	<2e-16
	Anti-Inflammatory Agent Dispensation Rates	0.007444	0.035407	0.21	0.833
	Paracetamol Dispensation Rates	−0.04697	0.039239	−1.2	0.231

	Dispersion parameter for beta family (): 0.221				
	AIC: 2666.5				
	BIC: 2693.2				
	logLik: 1326.3				
	deviance: 2652.5				
	df.resid: 329				

Females	(Intercept)	2.63888	0.19730	13.375	<2e-16
	**Anti-Inflammatory Agent Dispensation Rates**	**−0.09536**	**0.04626**	**−2.061**	**0.0393**
	Paracetamol Dispensation Rates	−0.05600	0.04461	−1.255	0.2094

	Dispersion parameter for beta family (): 0.148				
	AIC: 1760.5				
	BIC: 1787.2				
	logLik: 873.2				
	deviance: 1746.5				
	df.resid: 329				

**Table Legend (**Table 2**)**: The association analyses between regional year-wise suicide-related mortality (SRM) rates and anti-inflammatory agent dispensation rates were performed separately in females and males, applying zero-inflated generalized linear mixed effects models modeled on the general poison distribution. Year and an interaction term between proportional regional population and region consistuted random-intercept effects. Paracetamol dispensation rates were included as an independent fixed effects variable, and year and an interaction term between proportional regional population and region were included as random-intercept effects. Exposure variables (anti-inflammatory agent and paracetamol dispensation rates) were subjected to Blom-transformation prior to analyses. Odds ratios (exponentiation of β-values) and confidence intervals were calculated post-hoc. In the model for females, the anti-inflammatory agent variable exhibited OR = 0.9091 and the 95% CI (OR) was 0.8302, 0.9954. P-values <0.05 were considered significant (**bold**). Abbreviations: Coef, coefficient; OR, odds ratio; Std. Error, Standard Error; 95% CI, 95 percent confidence intervals.

**Table 3:** Associations between regional year-wise suicide-related mortality in 20–24-year-olds and anti-inflammatory agent dispensation rates – A generalized linear mixed effects model modeled on the beta binomial distribution.

		Coef.	Std. Error	z value	P-value
Females	(Intercept)	0.36104	0.22464	1.607	0.108
	**Anti-Inflammatory Agent Dispensation Rates**	**−0.33217**	**0.14942**	**−2.223**	**0.0262**
	Paracetamol Dispensation Rates	−0.09918	0.14618	−0.678	0.4975

	Dispersion parameter for beta family (): 1				
	AIC: 442.0				
	BIC: 464.9				
	logLik: 215.0				
	deviance: 430.0				
	df.resid: 330				

**Table Legend (**Table 3**)**: Suicide mortality rates in female 20-24-year-olds across the 21 Swedish regions during 2006–2021 were dichotomized based on the 25th percentile – whereby lower-quartile (Q1) SRM observations were compared to higher-quartile (Q2-Q4) observations. Generalized linear mixed effects models modeled on the beta binomial distribution and specifying the same random-intercept and fixed effects variables as in the main model were implemented. Paracetamol dispensation rates were included as an independent fixed effects variable, and region and year were included as random-intercept effects. Exposure variables (anti-inflammatory agent and paracetamol dispensation rates) were subjected to Blom-transformation prior to analyses. Odds ratios (exponentiation of β-values) and confidence intervals were calculated post-hoc. In the model for females, the anti-inflammatory agent exhibited OR = 0.7175 and the 95% CI (OR) 0.5353, 0.7173. P-values <0.05 were considered significant (**bold**).

Abbreviations: Coef, coefficient; OR, odds ratio; Std. Error, Standard Error; 95% CI, 95 percent confidence intervals.

### 3.3 Additional post-hoc analyses accounting for unspecified pain diagnosis rates

**Supplemental Fig. 8:**
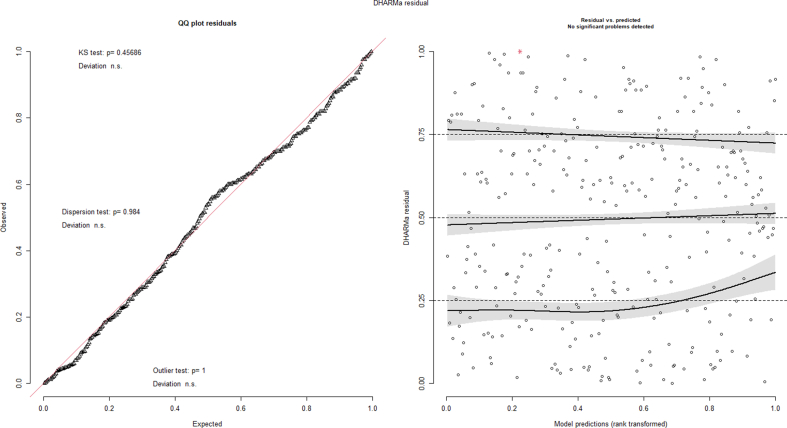
Title: QQ-plots residuals and residuals vs. predicted lines, post-hoc analysis incorporating unspecified pain (ICD-10 code: R52) diagnosis rates as covariate, main model

**Supplemental Fig. 9:**
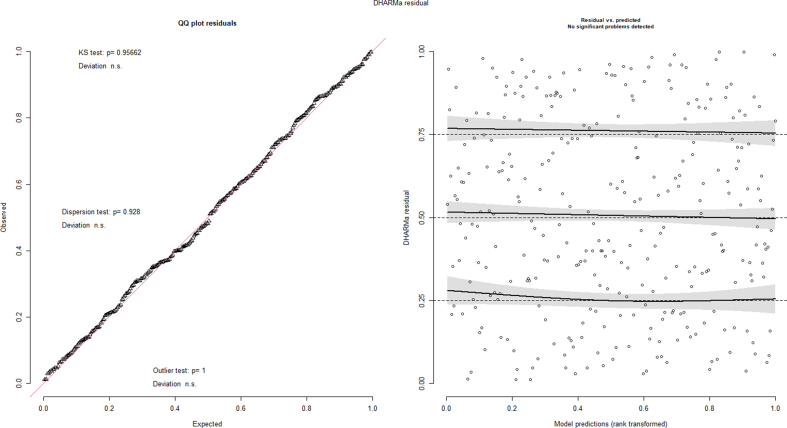
Title: QQ-plots residuals and residuals vs. predicted lines, post-hoc analysis incorporating unspecified pain (ICD-10 code: R52) diagnosis rates as covariate, dichotomized validation model

The association between NSAIDs prescription rates and SRM in females remained significant in post-hoc analyses performed with the inclusion of regional unspecified pain diagnosis rate (ICD-10 code:R52), both in the main model (β = −0.121 p = 0.0132) and the validation model of dichotomized data (OR = 0.990, p = 0.0376) (Supplemental Tables 1–2). Post-hoc analysis models incorporating regional unspecified pain diagnosis rates were tested for dispersion and heteroscedasticity, demonstrating no such signs (Supplemental Figs. 8–9).

## 4 Discussion

This study leverages real-world Swedish registry data across the 21 Swedish regions during 2006–2021 to investigate associations between regional year-wise suicide-related deaths and anti-inflammatory agent dispensation rates. The sample comprehensively encompassed all registered Swedish citizens that in the years 2006–2021 were aged 20-29-years-old and who died by a suicide-related event or were prescribed anti-inflammatory agents – in a sample encompassing 1657 deaths attributed to suicide (n = 1310) and damage events with unclear intent (n = 347). Drawing on regional and longitudinal differences for statistical inferences, we demonstrate that regional yearly dispensation rates for anti-inflammatory agents were negatively associated with SRM in 20–24-year-old females. The observational study design prevents causal inferences. Importantly, as anti-inflammatory agents are prescribed for several conditions associated with pain (which may confer increased suicide risk (Racine, 2018)), results may be burdened by selection bias. The independency of our results to regional year-wise paracetamol dispensation rates – also commonly prescribed for pain and associated conditions – contribute to reducing such concerns. Further alleviating such concerns is the robustness of the findings in post-hoc analyses accounting also for potential confound arising from regional year-wise diagnosis rates of unspecified pain conditions. These results add to the burgeoning literature indicating treatment gains from anti-inflammatory agents in the management of MDD (highly prevalent in retrospective studies of confirmed suicide deaths in adolescence and young adulthood (Bridge et al., 2006a)) and warrant the undertaking of further research efforts regarding the effects of NSAIDs in female young adults with MDD focusing on suicidality. If confirmed, NSAIDs may be a safe, inexpensive, and widely available treatment for the prevention of suicide deaths. Arguably, based on the confirmed efficacy of traditional antidepressants (Cipriani et al., 2018), it would seem reasonable to focus future research efforts on the potential role of NSAIDs as adjuvant treatment to traditional or more novel antidepressant agents.

A majority of anti-inflammatory prescription fills in this dataset pertained to non-selective NSAIDs (i.e. agents inhibiting both cyclooxygenase-1 (COX-1) and cyclooxygenase-2 (COX-2) receptors – compared to selective NSAIDs which only inhibit COX-2). Methodological constraints related to the aggregated nature of the dataset precluded investigation of the effects of selective and non-selective NSAIDs separately. Previous controlled trials have provided stronger support for the antidepressant effects of selective NSAIDs rather than non-selective agents (Köhler et al., 2014; Bai et al., 2020). A tentative hypothesis would be that the reductions in suicide-related mortality observed in our data set were conferred by reductions in depressive symptomatology from COX-2 inhibition. If correct, this would suggest that the findings from the present study hold relevance also for selective NSAIDs. However, any such assumptions must first be substantiated by confirmatory pharmacoepidemiological studies at the individual level. Given that combination treatment with NSAIDs and antidepressants is known to elevate risk of bleeding, which is a side effect exacerbated considerably by combination treatment with antidepressants (Masclee et al., 2014), the question of the comparable efficacy of non-selective vs. selective NSAIDs in a psychiatric context is not trivial. Large sample studies, albeit in populations substantially older than the present one, have demonstrated increased risk of intracranial haemorrhage (ICH) during the first 30 days of combined use of antidepressants and NSAIDs (Shin et al., 2015; Hou et al., 2021), a risk that has been indicated to be stronger in the case of non-selective agents (Hou et al., 2021). As aforementioned, limitations of previous research evaluating the effects of antidepressant treatment and NSAIDs have not allowed for thorough investigation of long-term adverse consequences – although reporting combination treatment as relatively safe (Köhler et al., 2014; Husain et al., 2017). Thus, there remains important safety aspects related to the concomitant use of SSRIs and NSAIDs in young adults that have yet to be fully elucidated. High-quality observational research with individual level registry data is warranted to further investigate efficacy and safety profiles of NSAIDs as adjuvant treatment to traditional antidepressants in young adults. Notably, it is possible that NSAIDs would confer lower risks of adverse outcomes as an adjuvant treatment to more novel non-SSRI antidepressant agents.

Substantial sex differences in suicide-related outcomes have been demonstrated in both adolescents (Bridge et al., 2006a) and adults (Naghavi, 2019b), implying distinct neurobiological mechanisms underlying suicidal behavior. Suicide mortality death rates are higher in males across all major western regions (Bridge et al., 2006b) – a pattern which was also observed in the present data set. The elevated suicide death rate in males may be related to increased prevalence of multiple risk factors in male adolescents, i.e., more lethal methods employed during suicide attempts, higher levels of comorbid substance abuse and affective psychopathology and elevated aggression (Naghavi, 2019b). The inability of the present study to discern significant suicide-related effects in stratified analyses of males may be related to study design limitations, biological differences, greater concordance between NSAID and paracetamol drug fills, or other factors. Data availability issues precluded the possibility to ascertain treatment adherence and explore the potential moderating role on the observed associations of clinical characteristics of individual recipients of anti-inflammatory agents, dosages and – importantly – the conditions precipitating anti-inflammatory agent prescription. Further, it was not possible to estimate the effect size of the observed association between NSAID prescription rates and lower SRM in females. Exposure variables did not satisfy normal distribution in the current study, necessitating Blom-transformation of data. Deriving meaningful estimates of co-variate effect sizes from analyses of Blom-transformed data may be unreliable, and were thus not reported. Well-designed prospective studies in thoroughly defined samples is necessary to clarify the potential moderating role of relevant clinical variables and to derive trustworthy estimates of the magnitude of potential effects of NSAID prescription on SRM and other suicide related behavior. Lastly, the study focused on young adults on the basis of the previously demonstrated association between inflammatory markers and depressive symptomatology (Kim et al., 2021) in this age group. Research in other age brackets would be necessary prior to generalization to other age groups.

In this retrospective nationwide observational study in Sweden covering the years 2006–2021 and comprehensively encompassing 1657 suicide-related deaths, regional year-wise anti-inflammatory agent dispensation rates in 20–24-year-old females were associated with lower suicide-related mortality. These results are in line with previous findings reporting that anti-inflammatory agents may be beneficial as adjunctive treatments for depression (a substantial contributor to suicide deaths). The causal establishment of such agents to depression and suicidality has, however, not been substantiated. Thus, these and previous findings on this topic warrant the conduct of further research on the potential role of anti-inflammatory agents as adjunctive treatments to antidepressants in at-risk populations. There is a lack of sound evidence to support the long-term safety aspects of combined NSAID and SSRI treatments (with indications of increased risks of serious adverse events). Targeted research is warranted to further elucidate long-term safety profiles of NSAIDs as adjuvant treatments to traditional antidepressants in young adults. Studying NSAIDs as an adjunctive treatment to novel non-SSRI antidepressant agents could possibly confer lower risks of adverse outcomes. If a clinically meaningful effect is confirmed, non-steroid anti-inflammatory agents may be a safe, inexpensive, and widely available treatment for the prevention of suicide deaths.

## Funding statement

Jussi Jokinen received funding from the 10.13039/501100004359Swedish Research Council (grant no. 2020–01183). The funding source had no influence in conceptualization, data extraction, analysis or interpretation of results pertaining to this study.

## Ethics statement

As the study pertained to open-access data, provided by a Swedish governmental agency for knowledge dissemination purposes, no formal ethical permission from a regulatory agency was required according to the relevant laws and statutes. The research was conducted in accordance with the principles of the Helsinki declaration.

## Declaration of competing interest

The authors declare the following financial interests/personal relationships which may be considered as potential competing interests: Jussi Jokinen reports financial support was provided by 10.13039/501100004359Swedish Research Council. Jussi Jokinen reports a relationship with Janssen Pharmaceuticals Inc that includes: consulting or advisory.

## Data Availability

Open-access data was used. Steps to retrieve the dataset utilized and replicate analysis is described in the manuscript.
