# Supplementary material for: Inverse association of anti-inflammatory prescription fills and suicide-related mortality in young adults: Evidence from a nationwide study of Swedish regions, 2006–2021
Source: Brain Behav Immun Health. 2023 Jun 28;31:100665. doi: 10.1016/j.bbih.2023.100665 (PMC10328802; doi:10.1016/j.bbih.2023.100665)
Supplement: Multimedia component 1 [file mmc1.docx]

|  | **Supplementary Table 1. Post-hoc Associations between Regional Year-Wise Suicide-Related Mortality in 20-24-year-old females and Anti-Inflammatory Agent Dispensation Rates, Adjusted for Rates of Unspecified Pain Diagnoses** | | | | |
| --- | --- | --- | --- | --- | --- |
|  |  |  |  |  |  |
|  |  |  |  |  |  |
|  |  | **Coef.** | **Std. Error** | **z value** | **P-value** |
| Females | (Intercept) | 2.702 | 0.253 | 10.673 | <2e-16 |
|  | **Anti-Inflammatory Agent Dispensation Rates** | **-0.121** | **0.0488** | **-2.479** | **0.0132*** |
|  | Paracetamol Dispensation Rates | -0.0009 | 0.0028 | -0.327 | 0.744 |
|  | Unspecified Pain Diagnosis Rate (ICD-10 code: R52) | -0.0591 | 0.0481 | -1.23 | 0.219 |
|  |  |  |  |  |  |
|  | AIC: NA |  |  |  |  |
|  | BIC: NA |  |  |  |  |
|  | logLik: NA |  |  |  |  |
|  | deviance: NA |  |  |  |  |
|  | df.resid: 300 |  |  |  |  |
|  | **Table Legend (Supplementary Table X)**: The post-hoc association analyses between regional year-wise suicide-related mortality (SRM) rates and anti-inflammatory agent dispensation rates was performed in females, applying zero-inflated generalized linear mixed effects models modeled on the general poison distribution. Year and an interaction term between proportional regional population and region consistuted random-intercept effects. Paracetamol dispensation rates and regional year-wise unique number of patients with a registered R52 diagnosis code (Unspecified Pain) were included as independent fixed effects variable, and year and an interaction term between proportional regional population and region were included as random-intercept effects. Exposure variables (anti-inflammatory agent, paracetamol dispensation rates and number of patients registered with R52 diagnosis rates) were subjected to Blom-transformation prior to analyses. P-values < 0.05 were considered significant (bold). Abbreviations: NA, not available. | | | | |
|  |  |  |  |  |  |

|  | **Supplementary Table 2. Post-hoc Validation Associations between Regional Year-Wise Suicide-Related Mortality in 20-24-year-olds and Anti-Inflammatory Agent Dispensation Rates – A Generalized Linear Mixed Effects Model Modeled on the Beta Binomial Distribution, Adjusted for Rates of Unspecified Pain Diagnoses** | | | | |
| --- | --- | --- | --- | --- | --- |
|  |  |  |  |  |  |
|  |  |  |  |  |  |
|  |  | **Coef.** | **Std. Error** | **z value** | **P-value** |
| Females | (Intercept) | 0.349 | 0.259 | 1.348 | 0.1777 |
|  | **Anti-Inflammatory Agent Dispensation Rates** | **-0.355** | **0.1708** | **-2.08** | **0.0376*** |
|  | Paracetamol Dispensation Rates | -0.0099 | 0.1597 | -0.062 | 0.9505 |
|  | Unspecified Pain Diagnosis Rate (ICD-10 code: R52) | -0.0809 | 0.1634 | -0.495 | 0.6203 |
|  |  |  |  |  |  |
|  | Dispersion parameter for betabinomial family (): 1 |  |  |  |  |
|  | AIC: NA |  |  |  |  |
|  | BIC: NA |  |  |  |  |
|  | logLik: NA |  |  |  |  |
|  | deviance: NA |  |  |  |  |
|  | df.resid: 300 |  |  |  |  |
|  | **Table Legend (Supplementary Table Y)**: Suicide mortality rates in female 20-24-year-olds across the 21 Swedish regions during 2006-2021 were dichotomized based on the 25th percentile – whereby lower-quartile (Q1) SRM observations were compared to higher-quartile (Q2-Q4) observations. Generalized linear mixed effects models modeled on the beta binomial distribution and specifying the same random-intercept and fixed effects variables as in the main model were implemented, with the addition that an additional independent fixed effects variable was included, adjusting for the regional year-wise number of unique patients diagnosed with an unspecified pain diagnosis (ICD-10: R52). Paracetamol dispensation rates were included as an independent fixed effects variable, and region and year were included as random-intercept effects. Exposure variables (anti-inflammatory agent, paracetamol dispensation rates and unique number of R52 diagnoses) were subjected to Blom-transformation prior to analyses. Odds ratios (exponentiation of β-values) and confidence intervals were calculated post-hoc. In the model for females, the anti-inflammatory agent exhibited OR=0.701 and the 95% CI (OR) 0.501, 0.979. P-values < 0.05 were considered significant (bold). Abbreviations: NA, not available | | | | |
